# Incidence, Clinical Characteristics and Attributable Mortality of Persistent Bloodstream Infection in the Neonatal Intensive Care Unit

**DOI:** 10.1371/journal.pone.0124567

**Published:** 2015-04-15

**Authors:** Jen-Fu Hsu, Shih-Ming Chu, Chiang-Wen Lee, Pong-Hong Yang, Reyin Lien, Ming-Chou Chiang, Ren-Huei Fu, Hsuan-Rong Huang, Ming-Horng Tsai

**Affiliations:** 1 Division of Pediatric Neonatology, Department of Pediatrics, Chang Gung Memorial Hospital, Taoyuan, Taiwan; 2 Division of Neonatology and Pediatric Hematology/Oncology, Department of Pediatrics, Chang Gung Memorial Hospital, Yunlin, Taiwan; 3 College of Medicine, Chang Gung University, Taoyuan, Taiwan; 4 Department of Nursing, Division of Basic Medical Sciences, and Chronic Diseases and Health Promotion Research Center, Chang Gung University of Science and Technology, Chia-Yi, Taiwan; 5 Research Center for Industry of Human Ecology, Chang Gung University of Science and Technology, Taoyuan, Taiwan; MUMC+ Academic Medical Center Maastricht, NETHERLANDS

## Abstract

**Background:**

An atypical pattern of neonatal sepsis, characterized by persistent positive blood culture despite effective antimicrobial therapy, has been correlated with adverse outcomes. However, previous studies focused only on coagulate-negative staphylococcus infection.

**Methods:**

All episodes of persistent bloodstream infection (BSI), defined as 3 or more consecutive positive blood cultures with the same bacterial species, at least two of them 48 hours apart, during a single sepsis episode, were enrolled over an 8-year period in a tertiary level neonatal intensive care unit. These cases were compared with all non-persistent BSI during the same period.

**Results:**

We identified 81 episodes of persistent BSI (8.5% of all neonatal late-onset sepsis) in 74 infants, caused by gram-positive pathogens (n=38, 46.9%), gram-negative pathogens (n=21, 25.9%), fungus (n=20, 24.7%) and polymicrobial bacteremia (n=2, 2.5%). Persistent BSI does not differ from non-persistent BSI in most clinical characteristics and patient demographics, but tends to have a prolonged septic course, longer duration of feeding intolerance and more frequent requirement of blood transfusions. No difference was observed for death attributable to infection (9.8% vs. 6.5%), but neonates with persistent BSI had significantly higher rates of infectious complications (29.6% vs. 9.2%, *P* < 0.001), death from all causes (21.6% vs. 11.7%, *P* = 0.025), and duration of hospitalization among survivors [median (interquartile range): 80.0 (52.5-117.5) vs. 64.0 (40.0-96.0) days, *P* = 0.005] than those without persistent BSI.

**Conclusions:**

Although persistent BSI does not contribute directly to increased mortality, the associated morbidities, infectious complications and prolonged septic courses highlight the importance of aggressive treatment to optimize outcomes.

## Introduction

Neonatal bloodstream infection (BSI) is the most common cause of mortality and morbidity after extremely preterm infants have survived the perinatal complications in the neonatal intensive care unit (NICU) [1–3]. The clinical presentation of neonatal BSI varies from mild symptoms to serious and life-threatening disease [4–6]. There is an entity that protracted recovery of pathogens from normally sterile body sites, correlating with prolonged inflammatory response and possibly adverse outcomes [7–11]. Persistent BSI has been reported during the past three decades, with rates ranging from 5% to 20% of neonatal late-onset sepsis [7–11]. Existing evidence suggests that persistent coagulase-negative *Staphylococcus* (CoNS) bacteremia is remarkable for thrombocytopenia and a substantial proportion of morbidities [11,12].

This atypical pattern of neonatal sepsis characterized by persistent BSI has been related with sequestered focal infection, contaminated indwelling devices, or result of biofilm formation [8,11]. In addition to persistent CoNS bacteremia, there is a paucity regarding other pathogens that remain positive for more than 3 days despite appropriate antibiotic treatment [8]. Besides, persistent fungal BSI is often ignored [13–15], and we do not know whether effective antibiotics should be modified when the blood culture remains positive with persistent clinical symptoms. The aim of the present study was to describe the clinical and laboratory profiles of neonates with persistent BSI in our NICU and to determine which risk factors were associated with the unusual syndrome.

## Patients and Methods

### Study setting, design and population

We reviewed the medical records of all neonates who were admitted to the NICU of Chang Gung Memorial Hospital (CGMH), a university-affiliated teaching hospital in Northern Taiwan with a total of 47 beds equipped with mechanical ventilators and 58 beds with special nurseries, and had persistent late-onset BSI (blood culture drawn >3 days of age) between July 1, 2004 and June 30, 2012. Infants with two blood cultures with different susceptibilities were excluded from the study. The study was approved by the Institutional Review Board of CGMH, with a waiver of informed consent. However, all patient records/information was anonymized and de-identified prior to analysis.

Demographical, clinical and microbiological variables at the onset of septic episode (defined as the time of the first obtained positive blood culture) were reviewed from our medical records. The presence of known or suspicious risk factors for persistent BSI [8–11] were also reviewed and included: invasive intubation or use of ventilator, use of central venous catheter (CVC), total parenternal nutrition (TPN) and/or intrafat. Because the septic conditions of each episode of BSI may fluctuate from one day to another, severity of illness was scored using the neonatal therapeutic intervention scoring system (NTISS) [16], with variables taken at the most severe day during the whole course of a BSI episode.

### Microbiological methods and antibiotic policy

Blood cultures were requested at the discretion of the attending physician and processed in the microbiology laboratory of our institution by the Bactec 9240 culture system. Identification of all causative pathogens was performed using standard microbiologic methods. Antibiotic susceptibility patterns were determined according to methods recommended by the National Committee for Clinical Laboratory Standards Institute (CLSI) for disk diffusion method and categorical assignment was carried out using CLSI breakpoints [17].

In our NICU, empirical antibiotics were prescribed for the coverage of both Gram-positive and Gram-negative organisms, usually oxacillin or vancomycin plus cefotaxime or gentamicin, once neonatal BSI was suspected. Modification of antimicrobial regimens would be made by the attending physician’s discretion, mostly according to the results and antibiotic susceptibility patterns of blood cultures. Blood cultures and laboratory evaluation were repeatedly performed in patients with persistent clinical manifestations, doubted culture results or progressive clinical deterioration, according to the discretion of attending physicians.

### Definitions

Persistent BSI was defined as 3 or more consecutive positive blood cultures with at least two of them 48 hours apart during a single sepsis episode. Otherwise, the episode of sepsis was considered as non-persistent BSI. Criteria from the Centers for Disease Control and Prevention were applied to define neonatal bacteremia [18]. All comorbidities of prematurity, including respiratory distress syndrome (RDS), intraventricular hemorrhage (IVH), bronchopulmonary dysplasia (BPD), and necrotizing enterocolitis (NEC) were based on the latest updated diagnostic criteria in the standard textbook of neonatology [19]. All concurrent infectious focus, including NEC, ventilator associated pneumonia (VAP), catheter-related bloodstream infection (CRBSI) or meningitis were also recorded and based on strict diagnostic criteria of Centers for Disease Control and Prevention and previous official publications [18,20,21]. Empirical antibiotic therapy was considered inappropriate if the treatment regimen did not include at least one antibiotic active *in vitro* against the infecting pathogens within 24 hours of blood culture collection.

For neonates with more than one episode of BSI, if the same pathogen was identified after a 14-day course of appropriate antibiotic therapy or at least one negative blood culture, or if a different pathogen was identified from a subsequent culture 7 days after the first one, it was considered as a new episode of BSI. If more than one pathogen were identified from a single set of blood culture or from different sets within a 72-hour period, it was defined as a polymicrobial BSI episode [22,23]. Infectious complications were defined as a newly onset infectious focus, venous thrombosis, or vegetation which is directly related to the episode of BSI or major organ dysfunction within one week after onset of the BSI. For patients who died during hospitalization, the cause of death was recorded according to the clinician’s presumption, and death was considered to be related to the BSI if one of the following was present: death within 3 days after the last positive blood culture of a single sepsis episode, death occurring before resolution of the signs and symptoms of bacteremia or fungemia, or autopsy finding indicated infection as the cause of death.

### Statistical analysis

All statistical analyses were performed with SPSS version 17.0 for Windows (SPSS Inc., Chicago, IL, USA). Results for continuous variables are presented as mean (± standard deviation). Univariate analysis was performed to identify significant differences in mean variables between the two study groups. Analysis of variance was used for continuous variables, and two-tailed Fisher’s exact test or Pearson’s chi-square test for categorical variables. For continuous variables with a non-Gaussian distribution, the nonparametric Mann-Whitney test was used. Statistical significance was defined as a *p* value of <0.05.

## Results

During the study period, 948 episodes of BSI at >3 days of age in 715 neonates were identified; of them, 81 episodes occurring in 74 patients were persistent (8.5% of all NICU late-onset BSI) and comprised the study population. Six patients had two episodes of persistent BSI, and one patient had three episodes of persistent BSI. The median duration of BSI in the persistent group was 3 days (range 2–18 days). In nearly half of the persistent BSIs, the causative pathogens were gram-positive organisms (n = 38, 46.9%), followed by gram-negative bacilli (n = 21, 25.9%), fungemia (n = 20, 24.7%) and polymicrobial bacteremia (n = 2, 2.5%). (Table 1)

**Table 1:** Comparison of distribution of bacterial pathogens between persistent and non-persistent bloodstream infection (BSI) in infants hospitalized in the neonatal intensive care unit.

	Persistent BSI n (%)	Non-persistent BSI n (%)
**Total episodes**	81 (100)	867 (100)
**Gram-positive organism**	38 (46.9)	506 (58.4)
Coagulase-negative *Staphylococcus*	19 (23.5)	356 (41.1)
*Staphylococcus aureus*	16 (19.8)	97 (11.2)
*Enterococcus* species	1 (1.2)	25 (2.9)
*Group-B streptococcus*	1 (1.2)	25 (2.9)
Others	1 (1.2)	3 (0.3)
**Gram-negative organism**	21 (25.9)	289 (33.3)
*Klebsiella* spp.	5 (6.2)	109 (12.6)
*Escherichia coli*	3 (3.7)	72 (8.3)
*Enterobacter* spp.	3 (3.7)	38 (4.4)
*Pseudomonas aeruginosa*	3 (3.7)	12 (1.4)
*Acinetobacter baumannii*	3 (3.7)	38 (4.4)
*Serratia marcescens*	2 (2.5)	9 (1.0)
Others*	2 (2.5)	11 (1.3)
**Fungus**	20 (24.7)	32 (3.4)
**Polymicrobial microorganisms** ^#^	2 (2.5)	40 (4.6)

*Including *Citrobacter freundii* (3), *Stenotrophomonas maltophilia* (3), *Hafnia alvei* (2), *Neisseria Meningitidis* (2), *Chryseobacterium meningoseptium* (1) *Morganella morganii* (1) and *Flavobacterium* (1)

^#^Indicating two or more microorganisms were recovered from the same blood culture set

There were no significant differences regarding demographic and clinical characteristics between persistent and non-persistent BSIs (Table 2), except for the higher rate of sequelae after operation for gastrointestinal pathology noted in neonates with persistent BSI. Although infants with persistent BSI were smaller and less mature at birth than the non-persistent infants, it did not reach significant difference. The presence of CVCs and an endotracheal tube, and the mean days of permanence time of CVC were also all comparable between neonates with persistent BSI and those with non-persistent BSI (Table 2). The presenting features of persistent BSIs when compared with non-persistent BSIs are described in Table 3. Most laboratory results were comparable between persistent BSIs and non-persistent BSIs, but anemia, thrombocytopenia, coagulopathy and metabolic acidosis were more frequently observed in episodes of persistent BSI. The illness severity (scored by NTISS) was also significantly higher in persistent BSIs than non-persistent BSIs, although the rates of hypotension and disseminated intravascular coagulopathy were comparable between these two groups.

**Table 2:** Comparison of demographic characteristics between persistent and nonpersistent bloodstream infections (BSIs) in infants hospitalized in the neonatal intensive care unit.

Characteristics	Persistent BSI (n = 81 episodes)	Non-persistent BSI (n = 867 episodes)	*P* value
Birth weight (g), median (IQR)	1210.0 (863.0–2410.5)	1280 (900.0–1975.0)	0.790
Gestational age (weeks), median (IQR)	29.0 (27.0–35.0)	30.0 (27.0–34.0)	0.749
Gender, (male/female), n (%)	44 (54.3)/37 (45.7)	456 (52.6)/411 (47.4)	0.816
Community-acquired/nosocomial*, n (%)	0 (0)/ 81 (100)	13 (1.5)/ 854 (98.5)	0.617
Day of life at onset of bacteremia (day), median (IQR)	30.0 (17.5–59.5)	28.0 (17.0–53.0)	0.418
Underlying chronic conditions, n (%)			
Congenital anomalies**	4 (5.6)	51 (5.9)	0.915
Neurological comorbidity, congenital or acquired	12 (19.4)	112 (12.8)	0.606
Cardiovascular			
Complicated congenital heart disease	5 (6.9)	30 (4.1)	0.213
Acyanotic heart disease with heart failure	0 (2.8)	21 (1.8)	0.157
Respiratory			
Bronchopulmonary dysplasia	32 (44.4)	335 (38.5)	0.318
Pulmonary hypertension and/or cor pulmonale	3 (4.2)	25 (3.2)	0.677
Renal^&^	6 (8.3)	32 (3.7)	0.063
Gastrointestinal			
Congenital GI pathology	6 (8.3)	52 (6.0)	0.439
Sequelae after operation of GI pathology^#^	7 (9.7)	30 (3.4)	0.018
Procedure or medical devices at onset of BSI, n (%)			
On high frequency oscillator ventilator	8 (11.1)	51 (5.9)	0.121
Intubation with mechanical ventilator	43 (53.1)	360 (41.5)	0.060
Presence of central venous catheter	72 (88.9)	727 (83.8)	0.197
Days of central venous catheter (mean ± SD)	18.9 ± 6.2	18.5 ± 5.2	0.535
Use of TPN and/or intrafat	56 (77.8)	647 (74.4)	0.576
Sequence of bloodstream infection, n (%)			0.787
First episode	60 (74.1)	657 (75.8)	
Recurrent episode	21 (25.9)	210 (21.3)	

*An episode of bloodstream infection detected in a neonate who had been discharged from the hospital and admitted due to young infant fever was considered to be community-acquired.

**Included all documented and undocumented syndrome, chromosome anomalies, genetic or metabolic disorder, but not simple cleft palate or polydactyly.

^&^Including congenital nephrotic syndrome, renal failure requiring hemodialysis and IgA nephropathy

^#^Including short bowel syndrome, GI pseudo-obstruction, adhesion ileus, and chronic malnutrition

**Table 3:** Comparison of clinical manifestations, treatment and outcomes between persistent and nonpersistent bloodstream infection (BSI) in infants hospitalized in the neonatal intensive care unit.

	Persistent BSI (n = 81 episodes)	Nonpersistent BSI (n = 867 episodes)	*P* value
Clinical manifestations			
Prolonged feeding intolerance (>3 days)	49 (60.5)	288 (33.2)	<0.001
Coagulopathy and/or GI bleeding	37 (45.7)	206 (23.8)	<0.001
Disseminated intravascular coagulopathy	10 (12.3)	74 (8.5)	0.303
Septic shock	20 (24.7)	142 (16.4)	0.064
Laboratory characteristics			
Leukopenia (WBC count <4,000/uL)	17 (21.0)	143 (16.5)	0.351
Leukocytosis (WBC count >20,000/uL)	27 (33.3)	240 (27.7)	0.302
WBC shift to left (immature WBC ≥20% total WBC)	10 (12.3)	106 (12.2)	0.978
Anemia (hemoglobin <11.0 mg/dL)	43 (53.1)	333 (38.4)	0.012
Thrombocytopenia (platelet <80,000/uL)	40 (49.4)	294 (33.9)	0.007
C-reactive protein^&^ (mg/dL), median (IQR)	40.7 (15.2–104.2)	32.8 (12.1–75.8)	0.293
Metabolic acidosis requiring jusomin replacement	23 (28.4)	160 (18.5)	0.039
NTISS score at most severe day of bacteremia, mean ± SD	18.0 ± 4.7	16.9 ± 4.6	0.034
Treatment modalities			
Appropriate antibiotics within 24 hours after bacteremia onset	41 (50.6)	644 (74.3)	<0.001
Removal of CVC within 3 days after bacteremia onset	27/77 (35.1)	294/829 (35.5)	0.944
Requirement of blood components transfusion	51 (63.0)	393 (45.2)	0.001
Required intubation/ventilator support with HFOV	43 (53.1)/ 12 (14.8)	360 (41.5)/ 84 (9.7)	0.060/ 0.175
Outcomes			
Infectious complications*	24 (29.6)	80 (9.2)	<0.001
Sepsis attributable mortality	8 (9.8)	56 (6.5)	0.245
Overall in-hospital mortality	16/74 (21.6)	75/641 (11.7)	0.025
Duration of hospitalization^#^	80.0 (52.5–117.5)	64.0 (40.0–96.0)	0.005

All data were expressed as number (percentage %), unless indicated otherwise; WBC: white blood cell; NTISS: Neonatal Therapeutic Intervention Scoring System; IQR: interquartile range; HFOV: high frequency oscillatory ventilator; CVC: central venous catheter

^&^CRP normal range: <5 mg/dL

*Infectious complications were defined as a newly infectious focus or persistent organ dysfunction which occurred within one week and directed related to bloodstream infection, but not concurrently at onset of bloodstream infection

^#^For 74 neonates with persistent BSI and 641 neonates without persistent BSI

Approximately half of persistent BSI episodes were treated with inappropriate antibiotics initially, but the therapeutic regimens were altered according to the susceptibility panel of the isolate as soon as it was reported. Persistent BSIs were associated with longer duration of feeding intolerance, and required more blood transfusions than non-persistent BSIs. Although similar percentage of CVCs was removed within three days in the group of persistent BSI, 58.4% of all CVCs in neonates with persistent BSI were then changed due to persistence of bacteremia. The sepsis attributable mortality rate was comparable between neonates with persistent and non-persistent BSIs, but neonates with persistent BSI were associated with a significantly higher rate of infectious complications (29.6% vs. 9.2%, *p* <0.001), death from all causes (21.6% vs. 11.7%, *P* = 0.025), and longer duration of hospitalization among survivors [median (interquartile range): 80.0 (52.5–117.5) vs. 64.0 (40.0–96.0) days, *P* = 0.005] than those without persistent BSI.

Among 81 episodes of persistent BSI, 69 (85.2%) had modification of therapeutic antibiotic regimens. After initiating appropriate antibiotics in 41 episodes of inadequately treated BSI, 9 (21.9%) episodes still had positive blood cultures. The physicians substituted teicoplanin for vancomycin (n = 13), carbapenem (n = 7) or cefotaxime (n = 5) for 3^rd^ generation cephalosporin or aminoglycoside, caspofungin for persistent fungemia (n = 11) and penicillin for vancomycin in one case of late-onset group B streptococcus bacteremia. 5 infants still died within one week even though antibiotics had been modified. Of the remaining 12 episodes without modification of therapeutic antibiotics, 3 infants died of infection after 4, 5 and 8 days of persistently positive blood cultures and others resolved after removal of infected catheters (n = 5), spontaneously (n = 2) and surgery (n = 2).

In the group of persistent BSI, the final adverse outcome or development of focal complications appeared to correlate with the duration of BSI. Among those with positive cultures for 3 days or less (n = 44, 54.3% of all persistent bacteremia), removal of infected catheters (n = 9, 20.5%), administrations of effective antibiotics (n = 24, 54.5%), or both (n = 10, 22.7%) resulted in eradication of the pathogens from bloodstream, and relatively benign courses were noted in these episodes. Only one case of persistent *Stenotrophomonas maltophilia* bacteremia died within 3 days. In the contrary, persistent BSI with duration of more than 4 days (n = 37, 45.7%) was associated with a significant high rate of focal complication or death. Septic hip, abdominal wall cellulitis, ventriculitis and pneumonia occurred in infants with persistent BSI for more than 3 days. Seven infants died of infection with catheter *in situ* after 5 to 13 days of persistently positive cultures (n = 4), focal complications (n = 2) and a superimposed sepsis (n = 1). Besides, fungal infection (n = 16, 43.2%) accounted for nearly half of persistent BSI episodes that lasted longer than 3 days.

For neonates with focal infectious complications after persistent BSI (n = 24, 29.6%), 13 (54.2%) had eradication of bloodstream pathogens and symptoms resolved after modifications of therapeutic antibiotics and removal of infected catheters. Five patients received surgery, and three died of infection. Three neonates had very long period of positive blood cultures (7, 13, and 18 days, respectively) and one of them with infected vena cava thrombus was followed up to two months for resolution of the thrombus.

## Discussion

We evaluated the clinical and microbiological profile of a more aggressive pattern of neonatal sepsis, characterized by persistent BSI with unresolved signs of septicemia despite antibiotic therapy. The high rate of indwelling CVCs at the diagnosis of bacteremia and more than half were treated with CVCs *in situ* rather than removal may contribute to the persistent positive blood culture [24,25]. We found the important role of removing the infected catheter in resolution of persistent BSI, which has been proven to reduce the mortality and morbidity rate [25,26]. Moreover, adding caspofungin and substituting more broad-spectrum antibiotics for aminoglycoside or 3^rd^ generation cephalosporin in persistent gram-negative BSIs were also important options towards the resolution of persistent BSI.

The incidence of persistent BSI reported by previous studies was much higher than our series. For example, 16% to 40.5% of CoNS bacteremia were reported to be persistent in previous studies [7,9,11,27]. However, their definitions of persistent BSI were different from ours: Chapman et al [7] applied recovery of CoNS >24 hours, Linder et al [27] defined recovery of CoNS >72 hours after adequate antibiotic therapy was begun and Patrick et al [11] used persistent bacteremia for positive blood culture of more than 6 days. Some other studies used the definition of 3 or more consecutive positive blood cultures with at least 2 of them 48 hours apart during a single sepsis episode after start of appropriate treatment [28,29]. Different definitions of persistent BSI absolutely affect the incidence, clinical characteristics, and results of this entity. We applied the most common definition of persistent BSI [8–10], which encompassed “persistence despite appropriate antibiotic therapy” and excluded the possibility of contaminants.

In our cohort, approximately half episodes of persistent BSI were related to initial inadequate antibiotics, but all of them had received an effective antimicrobial agent at least at the time of the last positive blood culture. We found delayed appropriate antibiotics may contribute to infectious complications instead of mortality [6], and the pathogens were easily eradicated once effective antibiotics were administrated. It was rarely necessary to use rifampin in our persistent CoNS or methicillin-resistant Staphylococcus aureus (MRSA) bacteremia [30]. Although we substituted teicoplanin for vancomycin in several episodes of persistent BSIs, vancomycin-resistant enterococci was rarely encountered. The resolutions of these persistent BSIs may result from removal of infectious focus or other treatments. Therefore we did not observe a significantly increased mortality in persistent CoNS or MRSA BSI, a finding that is consistent with several studies that concluded these gram-positive persistent BSI may be associated with a benign course [9,31].

Previous studies have concluded the association between focal suppurative complications and persistent BSI in the NICU infants, especially caused by non-CoNS pathogens [7,32,33]. Chapman et al [7] proposed that focal complications may possibly be the cause or the result of persistent infection. Although we have clarified the presences of concomitant infectious focus and infectious complications, some occult foci cannot be completely excluded. For example, the observed temporal relationship suggests that forearm cellulitis in one non-CoNS infant developed even after appropriate antibiotics were administered, but it is possible that the elbow was the site of a slowly evolving infection that gave rise to bacteremia and overlying cellulitis. Other reported short-term morbidities included thrombocytopenia, feeding intolerance, respiratory failure and chronic lung disease [11,12].

While some studies suggested that persistent BSI does differ from non-persistent BSI in some clinical characteristics and basic patient demographics [9,27], our results were different from theirs that the presences of CVCs and an endotracheal tube were not significantly associated with the development of persistent BSI. Based on our cohort, fungemia was the most vulnerable to become persistent and difficult to be eradicated, especially when systemic antifungal treatment was delayed [34]. In 21% of all fungal BSIs, persistent fungemia was still identified though it had been treated with fluconazole for more than 3 days, and we found caspofungin therapy was effect, safe and well-tolerated as an alternative therapy for persistent fungemia, a finding that is consistent with previous studies [35].

Except for persistent fungemia, approximately 46.9% of persistent BSIs were characterized with a relatively benign course; severe clinical features and abnormal laboratory findings were noted in only 38.3% of persistent BSIs. These relatively benign episodes may confound the insignificant comparisons between persistent and non-persistent BSIs. We found the remarkable characteristics of severe thrombocytopenia in our persistent bacteremia group, which was consistent with previous reports [9,10] and suggested this common feature was also present in non-CoNS persistent BSIs. A higher rate of anemia and coagulopathy was also noted, and the CRP level tended to vary widely between cases with benign courses and those with a fulminant course. Although persistent BSIs did not directly contribute to an increased mortality, it was associated with a higher risk of infectious complications, prolonged septic courses and a subsequent episode of sepsis, which accounted for the significantly higher rate of in-hospital mortality and longer duration of hospitalization.

There were some limitations in this study. Given its retrospective nature and the cohort came from a single center, therefore the general validity may be inevitably limited. Lacks of complete strain information and gene identification encoding surface protein B, biofilm or phenol-soluble modulins productions [8,10] were the weakness of this study. Lack of long-term follow up is another limitation of this study. However, this is the first study to enroll all persistent BSIs [7], including episodes of persistent fungal BSI, for analysis. The unselected nature of patients from extremely preterm to term-born infants, enrollment of all episodes of BSI, and extensive patient, infection and treatment information collected and analyzed are the strengths of the study.

In conclusion, persistent BSI represents an atypical pattern and more severe form of neonatal bacterema with longer duration of septic symptoms and higher rates of infectious complications. Although inappropriate antibiotics may contribute to part of the bacteremia persistence, it is possible that some strains of pathogens have acquired the capacity to persist which deserves further investigations.
